# Confinement Geometry Tunes Fascin-Actin Bundle Structures and Consequently the Shape of a Lipid Bilayer Vesicle

**DOI:** 10.3389/fmolb.2020.610277

**Published:** 2020-11-09

**Authors:** Yashar Bashirzadeh, Nadab H. Wubshet, Allen P. Liu

**Affiliations:** ^1^Department of Mechanical Engineering, University of Michigan, Ann Arbor, MI, United States; ^2^Department of Biomedical Engineering, University of Michigan, Ann Arbor, MI, United States; ^3^Department of Biophysics, University of Michigan, Ann Arbor, MI, United States; ^4^Cellular and Molecular Biology Program, University of Michigan, Ann Arbor, MI, United States

**Keywords:** actin, fascin, membrane deformation, encapsulation, giant unilamellar lipid vesicles

## Abstract

Depending on the physical and biochemical properties of actin-binding proteins, actin networks form different types of membrane protrusions at the cell periphery. Actin crosslinkers, which facilitate the interaction of actin filaments with one another, are pivotal in determining the mechanical properties and protrusive behavior of actin networks. Short crosslinkers such as fascin bundle F-actin to form rigid spiky filopodial protrusions. By encapsulation of fascin and actin in giant unilamellar vesicles (GUVs), we show that fascin-actin bundles cause various GUV shape changes by forming bundle networks or straight single bundles depending on GUV size and fascin concentration. We also show that the presence of a long crosslinker, α-actinin, impacts fascin-induced GUV shape changes and significantly impairs the formation of filopodia-like protrusions. Actin bundle-induced GUV shape changes are confirmed by light-induced disassembly of actin bundles leading to the reversal of GUV shape. Our study contributes to advancing the design of shape-changing minimal cells for better characterization of the interaction between lipid bilayer membranes and actin cytoskeleton.

## Introduction

Dynamics of actin filaments are coordinated to form different networks that regulate cellular functions by supporting and remodeling the plasma membrane (Fletcher and Mullins, 2010; Stricker et al., 2010), generating driving forces for cell motility and cell division, facilitating cell adhesion to extracellular matrix (Burridge et al., 1990), and spatially organizing cellular machineries (Matsudalra, 1991; Pollard and Borisy, 2003; Pollard and Cooper, 2009). The dynamic organization of actin networks is regulated by a number of accessory proteins that coordinate actin polymerization/de-polymerization and crosslink actin filaments to one another, to other cytoskeletal components, and to cellular membranes. Actin crosslinkers organize actin filaments into bundles and networks, and each type of actin crosslinker has specific attributes to regulate actin organization and subsequently cellular functionality (Matsudalra, 1991; Tseng et al., 2002), including cell locomotion. Given the diversity in crosslinkers and architecture of actin networks, it is important to understand how the assembly of actin networks induces morphological changes of cells and how multiple crosslinkers orchestrate cellular shape changes.

During cell motility, two distinct structures are formed at the leading edge of the cell: filopodia and lamellipodia. Filopodia is a protrusive organelle that together with lamellipodia drives cellular motility, particularly by functioning as a sensor to the external environment (Ridley et al., 2003; Adams, 2004). The prominent actin crosslinker in filopodia is fascin (Yamashiro et al., 1998). Fascin is a small crosslinker that forms linear, closely packed bundles from parallel filaments that, under specific circumstances, dynamically remodels the plasma membrane through the formation of filopodia (Otto et al., 1979; Small, 1988; Lewis and Bridgman, 1992; Svitkina et al., 2003). Prior *in vitro* reconstitution works have shown the mechanism of filopodia formation and the role of fascin as an actin filament crosslinker forming stiff linear actin bundles (Vignjevic et al., 2003; Yang et al., 2013). Using *in vitro* reconstitution on functionalized beads and supported lipid bilayers, research has demonstrated the transition of actin filaments from dendritic networks to bundled networks by the recruitment of fascin as actin filaments elongate (Haviv et al., 2006; Vignjevic et al., 2006; Lee et al., 2010). Furthermore, reconstituted dendritic actin networks on the surface of giant unilamellar vesicles (GUVs) have been shown to spontaneously generate filopodia-like protrusions that deform membranes in the absence of fascin (Liu et al., 2008; Simon et al., 2019). Moreover, research has also delved into how two crosslinkers, fascin and α-actinin, concurrently generate force to form filopodia and its subsequent effect in enhancing the mechanical response of the cell (Tseng et al., 2005). Recently, it was also demonstrated that the size of fascin and α-actinin allows them to self-sort *in vitro* when forming actin bundles by inhibiting the recruitment of one another (Winkelman et al., 2016).

Although actin crosslinkers such as fascin have been well-studied using various reconstitution assays previously, it remains unclear how different actin crosslinkers individually and synergistically construct actin bundle structures that generate forces to induce membrane deformation. Encapsulation of actin and accessory proteins inside membrane-enclosed systems such as GUVs allow for studying the orchestration of actin networks and their interaction with the lipid membrane in a cell-like compartment without the involvement of complex cell signaling pathways (Bashirzadeh and Liu, 2019). Encapsulated actin crosslinkers such as fascin, α-actinin, and filamin assemble actin into diverse structures (Honda et al., 1999; Abkarian et al., 2011; Bashirzadeh et al., 2020; Litschel et al., 2020). These structures have been shown to induce diverse GUV morphological changes (Honda et al., 1999; Limozin and Sackmann, 2002; Abkarian et al., 2011; Tsai and Koenderink, 2015; Litschel et al., 2020), and the shape changes were recently shown to be reversible by light-induced actin bundle disassembly (Litschel et al., 2020). Recent advances in encapsulation methods have been shown to be effective in generating GUVs encapsulating functional proteins (Majumder et al., 2019; Litschel et al., 2020). Reconstitution of actin networks inside GUVs has shown motor protein-induced membrane deformation by contracting membrane-associated actin cortex and rings (Maan et al., 2018; Litschel et al., 2020), but examples of such cellular reconstitution studies remain limited.

By confining actin polymerization and bundling process in GUVs, here we studied the formation of actin structures by fascin and their interaction with the lipid membrane. We found that membrane deformation patterns were correlated to the concentration of fascin. In addition, small GUVs increased bundle-bundle proximity, fascin-induced crosslinking at the GUV center, and the formation of protrusions akin to filopodia. Actin bundle-induced deformations could be reversed by actin de-polymerization using exposure to light. Light-induced actin bundle disassembly and restorative property of deformed membranes are further characterized as a function of actin bundle architecture. Moreover, we co-encapsulated fascin and α-actinin at different concentration ratios and studied changes in membrane deformation as a function of GUV size and GUV shape restoration in comparison to fascin alone. Our results highlight the feasibility of the confinement of a mixture of biopolymer networks for studying cytoskeleton-membrane interactions and cellular shape changes.

## Materials and Methods

### Preparation of Proteins and Reagents

We purified actin from rabbit skeletal muscle acetone powder (Pel-Freez Biologicals) as described previously (Pardee and Aspudich, 1982) or purchased it from Cytoskeleton Inc, United States. ATTO 488 actin was purchased from Hypermol Inc, Germany. α-Actinin was purchased from Cytoskeleton Inc. We purified fascin from *E. coli* as Glutathione-S-Transferase (GST) fusion protein. For purification, BL21(DE3) *E. coli* cells was transformed with pGEX-4T-3 (GE Healthcare) containing the coding sequences of fascin. Cells were grown at 37°C while shaking at 220 rpm until the OD_600_ reached 0.5–0.6. Protein expression was induced with 0.1 mM IPTG and cell culture was incubated at 24°C for 8 h. Cells were harvested by centrifugation at 4,000 × g for 15 min and washed with PBS once. Pellets were stored at −80°C until the day of purification. Cell pellets were resuspended in lysis buffer (20 mM K-HEPES pH 7.5, 100 mM NaCl, 1 mM EDTA, 1 mM PMSF) and ruptured by sonication. Cell lysates were centrifuged at 75,000 × g for 25 min and supernatants were loaded on a GSTrap FF 1 mL column (GE Healthcare) using an AKTA Start purification system (GE Healthcare) at a flow rate of 1 mL/min. The column was washed with 15 mL washing buffer (20 mM K-HEPES pH 7.5, 100 mM NaCl) and the protein was eluted with 5 mL elution buffer (washing buffer + 10 mM reduced L-glutathione). Purified fascin was dialyzed against 1 L PBS twice for 3 h and once overnight at 4°C. Protein concentration was calculated by UV absorption using predicted molar extinction coefficients (ExPasy) of 110,700 M^–1^cm^–1^. Proteins were concentrated with Centricon filters (Merck-Millipore) when needed and/or diluted to a final concentration of 1 mg/mL in PBS.

### Generation of GUVs

We modified continuous droplet interface crossing encapsulation (cDICE) technique for robust encapsulation in GUVs of various sizes (Abkarian et al., 2011; Bashirzadeh et al., 2020; Litschel et al., 2020). Briefly, a custom 3D-printed chamber is mounted on a benchtop stir plate and rotated at 1,200 rpm. An outer solution of 200 mM glucose (matched to the osmolarity of the inner solution) is pipetted into the chamber. Then, the lipid mixture (∼70% DOPC, 30% cholesterol, and a trace concentration of fluorescent lipid) in a mixture of silicone oil and mineral oil (4:1) is added. The lipid-oil solution forms an interface at the oil–water interface. Inner solution containing 5 μM actin including 10% ATTO 488 actin in polymerization buffer containing 50 mM KCl, 2 mM MgCl_2_, 0.2 mM CaCl_2_, and 4.2 mM ATP in 15 mM Tris, pH 7.5 and 5% OptiPrep, 0.25–2.5 μM fascin and/or 0.5–1.5 μM α-actinin were added into 700 μL of the lipid–oil mix and droplets were generated by pipetting up and down. GUVs were then generated by dispensing the droplets into the cDICE chamber. A lipid bilayer is formed when a second layer of lipid is acquired as the droplets cross the lipid/oil-outer solution interface.

### Bulk Fascin-Actin Bundle Assay

Five micrometers of monomeric actin with different concentrations of fascin were added to the actin polymerization buffer containing the same amounts buffer, salts, and ATP as those used for encapsulation experiments. Thirty minutes after polymerization and bundling at room temperature, Acti-stain 488 phalloidin was added (instead of using ATTO 488 actin) to stabilize actin bundles.

### Imaging and Image Processing

Following cDICE, GUVs were transferred to a 96-well plate for imaging. OptiPrep in the inner solution increases GUV density and accelerates sedimentation of GUVs onto the bottom of well plate. For imaging actin bundles in bulk, the solution containing actin bundles was confined between a cover slip and glass slide. All samples were imaged at least 1 hour after actin polymerization and bundling. An Olympus IX-81 inverted microscope equipped with a spinning disk confocal (Yokogawa CSU-X1), AOTF-controlled solid-state lasers (Andor Technology), and an iXON3 EMCCD camera (Andor Technology) was used for microscopy. Images were magnified by an oil immersion 60×/1.4 NA objective lens mounted on the microscope. Images were acquired by MetaMorph software (Molecular Devices). Fluorescence images of actin and lipids were taken with 488 nm and 561 nm laser excitation, respectively. Z-stack fluorescence confocal image sequence of lipids and actin were taken with a z-step size of 0.5 μm.

Images were processed using ImageJ/Fiji (Schindelin et al., 2012; Schneider et al., 2012; Litschel et al., 2020), SOAX (Xu et al., 2014, 2015), and MATLAB routines (Bashirzadeh et al., 2020). 3D images were created using 3D Project command in ImageJ/Fiji. Skeletonized bundles were generated by SOAX source code (Xu et al., 2014, 2015) following image-processing of z-stack image sequences using ImageJ/Fiji, complemented with the plugin Squassh (Rizk et al., 2014) from the MOSAIC ToolSuite update site (Litschel et al., 2020). MATLAB routines were developed to reconstruct generated SOAX text files as a USCF Chimera marker file, include a colormap for z coordinates, and save file as.cmm format for 3D visualization in USCF Chimera (Pettersen et al., 2004).

### Data Analysis

Actin bundle phenotypes and GUV shape changes were characterized from z-stack actin and lipid images. The diameter of a GUV was measured by line scan from GUV images. The percentages and probabilities of mid-plane bundle formation, network formation, filopodia-like protrusions, and GUV shape changes (membrane deformation and/or filopodia-like protrusions) were obtained by their count divided by the total number of GUVs with actin bundles (i.e. GUVs encapsulating fluorescent actin monomers with no sign of bundling activity were not counted). We calculated the persistence length of fascin-actin bundles using the skeletonized images of actin bundles by measuring their orientational correlation function as described previously (Bashirzadeh et al., 2020). Persistence length values are shown as average ± standard error of the mean of the data at each fascin concentration.

For probability and percentage measurements at least 2 independent experiments were conducted for each condition indicated. The reported *p* values are two-tail, unpaired two-sample student t-test assuming unequal variances.

## Results

### Actin Bundling by Fascin Reshapes GUVs to Different Geometries Yet Reversible by Light

**FIGURE 1:**
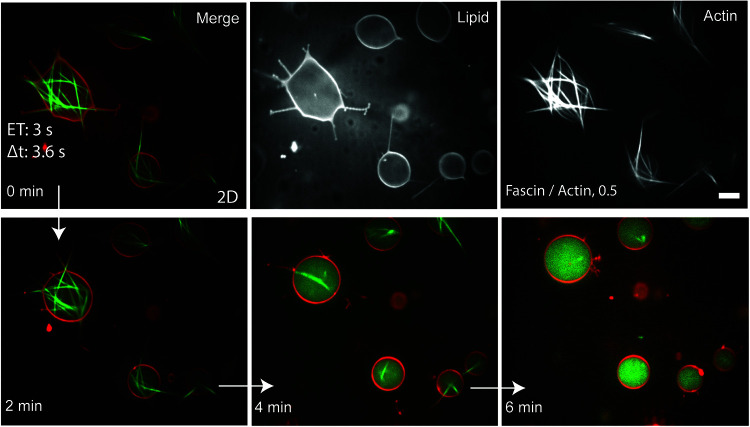
Actin **(top right)**, lipid **(top middle)**, and merged **(top left)** confocal images of actin bundles which assembled to deform GUVs and formed long filopodia-like membrane protrusions. Application of 488 nm light at high exposure times (3 s) resulted in the disassembly of actin bundles and reversal of GUV shape without affecting the integrity of the lipid bilayer. Images were captured at 1 frame/3.6 s. Fascin/actin, 0.5 (M/M). Scale bar, 10 μm.

We first encapsulated actin and fascin in actin polymerization buffer into GUVs to observe F-actin bundling activity in a membrane-enclosed environment. Fascin formed rigid actin bundles some of which were non-protrusive yet deformed the membrane while others were rigid enough to protrude into the membrane and formed long filopodia-like structures (Figure 1). Stabilized GUVs deformed by fascin bundles were restored to their spherical resting shape due to actin bundle disassembly under prolonged exposure to 488 nm light (Figure 1). In the absence of actin, prolonged light exposure has no impact on vesicle shapes at all. This confirms that bundle elongations act as the driving force for GUV shape changes, and they support and stabilize deformed GUVs. Although bundle disassembly under light exposure was accompanied by GUV shape reversal, the absence of bundle-membrane interactions in filopodia-like membrane protrusions and disassembly of actin filaments in their interior destabilized the now actin-free protruded membrane. After the disassembly of actin bundles, all GUVs maintained their spherical shape without rupturing (Supplementary Video S1).

### GUV Size-Dependent Amalgamation of Fascin-Bundles Contribute to Protrusion

**FIGURE 2:**
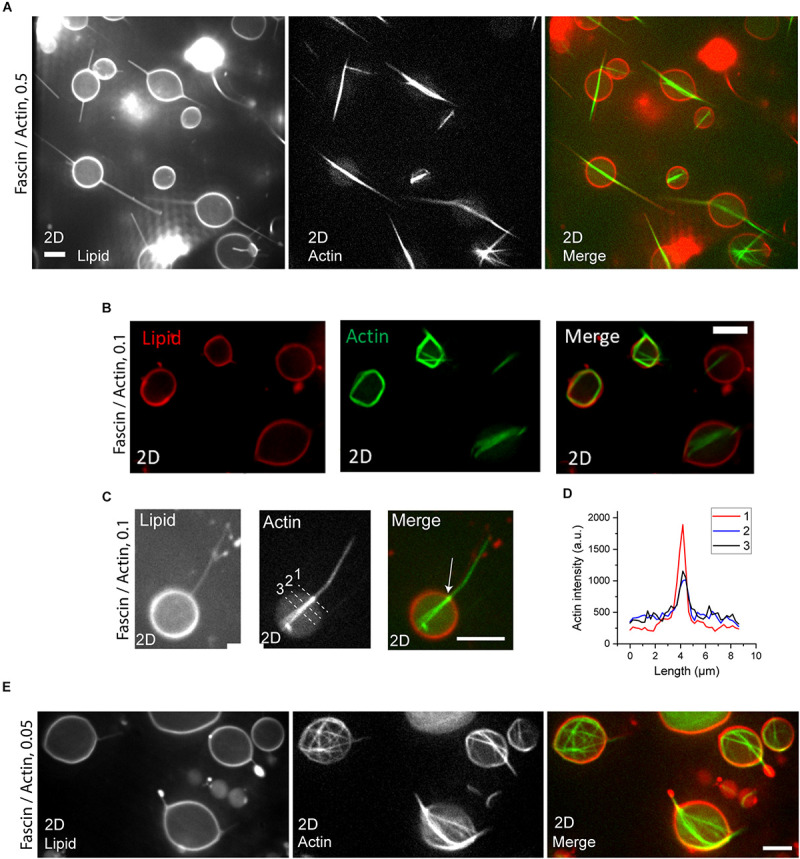
**(A,B)** Actin (left), lipid (middle), and merged (right) confocal images of actin bundles in small GUVs (diameter < 16 μm) at fascin concentrations of 2.5 μM **(A)** and 0.5 μM **(B)**. Actin, 5 μM. Scale bars, 10 μm. **(C)** Actin (left), lipid (middle), and merged (right) confocal images of a protruding actin bundle with aggregated actin at the point of initiation of membrane protrusion (arrow) at fascin concentration of 0.5 μM. **(D)** Actin fluorescence intensity profile across the dashed lines in **(C)**. **(E)** Actin (left), lipid (middle), and merged (right) confocal images of actin bundles in small GUVs (diameter < 16 μm) at a fascin concentration of 0.25 μM. Actin, 5 μM. Scale bar, 10 μm.

In order to test the influence of confinement geometry on actin bundling by fascin and interaction of bundles, we encapsulated fascin and actin at different molar ratios in a large population of GUVs with varying sizes. Fascin-bundles appeared as either networks or a single bundle in GUVs (Figure 2). At fascin/actin molar ratio of 0.5, fascin-bundles in small GUVs (7–16 μm diameter) mostly appeared as long bundles at the GUV midplane which protrude into the membrane to form long filopodia-like structures from both ends of the bundle. Bundles were straight and their lengths were consistent with those observed in bulk, some spanning beyond GUV diameter (Supplementary Figure 1) (Claessens et al., 2008; Breitsprecher et al., 2011). We find that fascin-to-actin molar ratio determines bundle length in our encapsulated system, as opposed to the concentration of actin and fascin in bulk experiments (Claessens et al., 2008). At fascin/actin molar ratio of 0.5 and 0.1, actin bundles in most GUVs appeared as an amalgamation of several actin bundles changing GUV shape from spheres to spindles (Figures 2A,B). Some GUVs, however, deformed into irregular shapes by crimped or protruded actin bundles the majority of which had lengths well above GUV diameter (Figures 2A,B). The majority of single bundles aligned horizontally with respect to the GUV surface and localized at the GUV equatorial plane. Actin localization was often more intense at the locus of membrane protrusion in GUVs (Figures 2C,D) which could possibly be an indication of membrane-induced actin bundling to facilitate protrusion (Liu et al., 2008). By decreasing fascin concentration to a molar ratio of 0.05, we rarely observed single bundles. Rather, the majority of actin bundles appeared to be forming networks in small GUVs (7–16 μm diameter) (Figure 2E).

**FIGURE 3:**
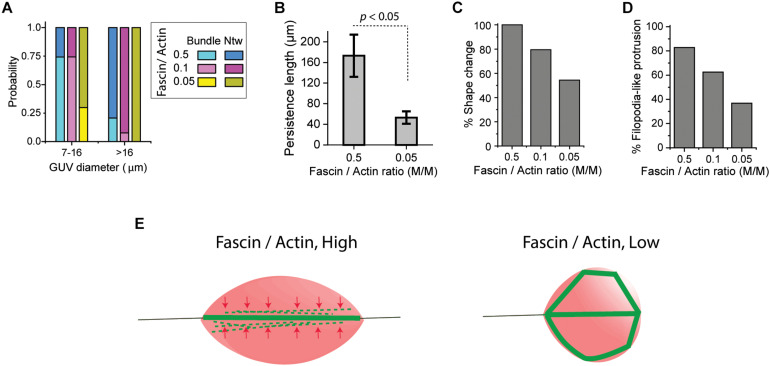
**(A)** Probability of the formation of a single actin bundle (Bundle) or networks (Ntw) in small GUVs (7–16 μm diameter) and large GUVs (>16 μm diameter) at actin concentration of 5 μM with different fascin concentrations indicated. Number of GUVs >30 per experiment per category; ≥2 independent experiments per fascin concentration. **(B)** The persistence length of actin bundles at two fascin concentrations. Actin, 5 μM; >18 bundles in 6 GUVs across two independent experiments were analyzed per fascin concentration. Error bar represents standard error of the mean. **(C)** The percentage of small GUVs (7–16 μm diameter) with shape changes (deform and/or protrude) by fascin-actin bundles at various fascin concentrations indicated. Number of GUVs > 30 per experiment per category; ≥2 independent experiments per fascin concentration. **(D)** The percentage of small GUVs (7–16 μm diameter) with filopodia-like membrane protrusions by fascin-actin bundles at different fascin concentrations indicated. Actin concentration is 5 μM. Number of GUVs > 30 per experiment per category; ≥2 independent experiments per fascin concentration. **(E)** Schematic representation of the formation of single actin bundles at high fascin concentrations (left) and actin bundle structures around the membrane at low fascin concentrations (right) in small GUVs (7–16 μm diameter). Small geometry of confinement increases the interaction of fascin-bundled actin filaments at high fascin concentrations to merge into a stiff protruding bundle at the GUV mid-plane. These bundles cause GUVs to deform into spindle-like vesicles with filopodia-like protrusions.

Analyzing GUV populations at three fascin molar ratios showed that indeed, the probability of the formation of a single actin bundle in small (7–16 μm diameter) GUVs was high (0.75) at fascin-to-actin ratios of 0.5 and 0.1 while network formation probability was high (0.8 and 0.92, respectively) in large (>16 μm diameter) GUVs at the same fascin molar ratios (Figure 3A). Increased fascin-mediated interaction between actin filaments being bundled in a small volume increase the chance of actin to merge into a single bundle. The balance of forces in and on GUVs possibly positions single bundles in the GUV equatorial plane. The resistance of long packed bundles against bending under the influence of membrane curvature could also contribute to the merging of actin bundles at the GUV mid-plane. Such resistance, described in terms of bundle persistence length, is highly influenced by crosslinker type and concentration (Claessens et al., 2006a; Takatsuki et al., 2014). The persistence length of encapsulated fascin-actin bundles were indeed increased from 53 ± 12 μm to 173 ± 41 μm by increasing fascin molar ratio from 0.05 to 0.5 which is significantly greater than the F-actin persistence length (∼14–17 μm) (Figure 3B) (Le Goff et al., 2002). Such increase is consistent with an increase in persistence length of fascin-actin bundles in bulk and motility assays values from ∼20 μm at molar ratio of 0.05 to ∼150 μm at molar ratio of 0.5 (Takatsuki et al., 2014). The increase in persistence length is an indication of the effect of encapsulation in merging nearby actin bundles with high flexural rigidity. Encapsulation of actin networks has also been shown to increase network stiffness by diminishing filament fluctuations (Claessens et al., 2006b). We found no relationship between the length and persistence length of the encapsulated actin bundles. While high persistence length of the elongated fascin-actin bundles favors merging and protrusion at the mid-plane of small GUVs, lowering fascin concentration and consequently bundle persistence length can induce the formation of kinked actin bundle structures around GUV periphery. Fascin at a molar ratio of 0.5 induced large-scale membrane shape changes in all small GUVs (Figure 3C), the majority of which had at least one filopodia-like membrane protrusion (Figure 3D). Fascin concentration here was high enough to induce GUV shape changes in all large GUVs with the majority of them appearing to have filopodia-like protrusions (not statistically analyzed). At a low fascin concentration, smaller portion of GUVs changed shape as expected due to lower bundle rigidity (Takatsuki et al., 2014) (Figures 3C,D). The schematic in Figure 3E illustrates bundle-bundle interactions at high fascin concentrations in small GUVs that could lead to the formation of a single bundle with increased rigidity at the GUV midplane thereby contributing to membrane protrusion. At low fascin concentrations, low persistence length and the absence of bundle-bundle interactions result in bundle buckling and/or bending around the membrane to form peripheral actin bundle structures.

### Fascin-Induced Filopodia-Like Protrusions Are Abrogated in the Presence of α-Actinin

**FIGURE 4:**
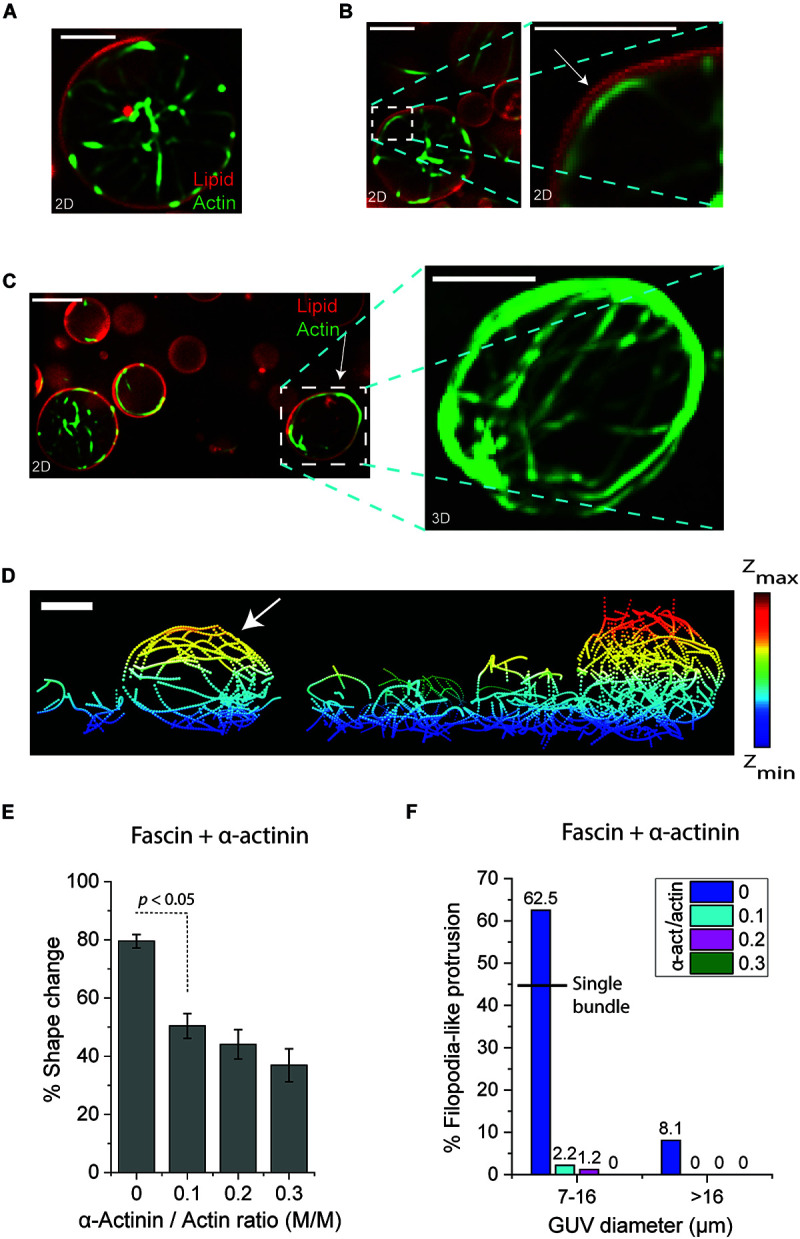
**(A–C)** Representative confocal merged images (actin:green, lipid:red) of GUVs encapsulating actin bundles in the presence of fascin and α-actinin. Actin bundles in the lumen of large GUVs do not deform GUVs **(A)**. Localized actin bundles **(B)** and meshwork **(C)** at or around the periphery deform GUVs (arrows). Fascin/actin, 0.1 (M/M). α-actinin/actin, 0.1 (M/M). Scale bars, 10 μm. **(D)** Skeletonized image of encapsulated actin bundles in **(C)**. Actin meshwork in the deformed GUV is indicated by arrow. Scale bar, 10 μm. **(E)** The percentage of small GUVs (7–16 μm diameter) with shape changes by fascin-actin bundles without and with α-actinin at various concentrations. Fascin/actin, 0.1 (M/M). Actin, 5 μM. Error bars indicate standard error of the mean. Number of GUVs > 30 per experiment per category; three independent experiments per category. **(F)** The percentage of GUVs with filopodia-like membrane protrusions formed by fascin-actin bundles without and with α-actinin at various concentrations in small (7–16 μm diameter) and large (>16 μm diameter) GUVs. The black line indicates the probability of filopodia-like protrusion in small GUVs encapsulating a single actin bundle. Fascin/actin, 0.1 (M/M). Actin, 5 μM. Number of GUVs > 30 per experiment per category; three independent experiments per category.

To explore how the flexibility of actin bundles can influence their interaction at the midplane of GUVs and subsequent GUV shape changes, we sought to co-encapsulate fascin and α-actinin with actin in GUVs. The persistence length of encapsulated α-actinin-actin bundles is lower than the persistence length of fascin-actin bundles measured here (Takatsuki et al., 2014; Bashirzadeh et al., 2020). This causes α-actinin-actin bundles to bend around the membrane and merge into an actin ring in cell-sized GUVs in order to minimize their elastic energy (Miyazaki et al., 2015) (Supplementary Figure 2A). Bending also increases the chance of actin bundle-membrane interactions and colocalization of actin filaments with the membrane (Supplementary Figure 2B). Encapsulation of fascin, α-actinin, and actin in GUVs caused a portion of actin bundles to bend at the GUV periphery (Figure 4A) some of which resulted in local (Figure 4B) or large-scale (Figure 4C) membrane deformations by forming an actin meshwork around the periphery (Figure 4D, arrow). Reversal of the GUV shape to its original spherical shape under exposure to 488 nm light was observed similar to that observed in the presence of fascin-only bundled actin (Figure 1, Supplementary Figures 3A–C, and Supplementary Videos 2–4). This confirmed that fascin-α-actinin actin bundles drove GUV shape changes and applied lateral forces to deform GUVs. Higher α-actinin concentration required higher exposure time indicating that the presence of α-actinin can suppress bundle disassembly under light exposure (Supplementary Figure 3D and Supplementary Video 5). This was confirmed by the observation that α-actinin-actin networks do not disassemble by 488 nm light (Supplementary Figure 3E and Supplementary Video 6). Further investigations are required to examine the influence of light on association/dissociation rate and bundling ability of α-actinin and fascin.

Although fascin-α-actinin-actin bundles could often deform GUVs, only around half of small (7–16 μm diameter) GUVs changed shape in the presence of α-actinin and fascin both at a molar ratio of 0.1. This was significantly lower compared to the number of GUVs deformed by fascin-actin bundles at a fascin molar ratio of 0.1 (50% < 80%) (Figure 4E). The portion of small GUVs which changed shape further decreased by increasing α-actinin molar ratio from 0.1 to 0.3 (37% < 50%) (Figure 4E). The majority of fascin-actin bundles at fascin molar ratio of 0.1 induced filopodia-like membrane–protrusions (62.5%) in small GUVs more than two-third of which was by protrusion of single bundles at the GUV midplane (Figure 4F). Addition of α-actinin at molar ratio of 0.1 abrogated the formation of fascin-induced filopodia-like membrane protrusions (2.2%) (Figure 4F). No filopodia-like structures were observed by increasing α-actinin concentration to a molar ratio of 0.3. Similar trend was observed in large GUVs. However, filopodia-like protrusions by fascin-actin bundles were also significantly lower in large (>16 μm diameter) GUVs than small (7–16 μm diameter) GUVs (8.1% < 62.5%) (Figure 4F). These results underscore the influence of actin crosslinkers and possibly a crosslinker competition mechanism for actin bundling on bundle persistence length and consequently GUV shape changes.

## Discussion

By cellular reconstitution of actin and actin crosslinking proteins in GUVs, we could recapitulate diverse membrane morphologies. We showed that both confinement geometry and crosslinker concentration influence bundle–bundle and bundle–membrane interactions in confinement thereby determining actin bundle architecture and GUV shape. These results highlight the influence of cell size and density of actin crosslinking proteins on the interaction of actin cytoskeleton and plasma membrane in small scale and the overall cellular structure in a larger scale. We speculate that depletion effects in small volume increase the chance of non-specific attractions between filaments and bundles which contribute to ATP hydrolysis-dependent actin polymerization and fascin-mediated bundling (Marenduzzo et al., 2006a,b; Miyazaki et al., 2015). We showed that high fascin concentration significantly contributed to the amalgamation of actin bundles into one protruding unit in small GUVs. Bundle persistence length was shown to sharply increase by increasing the concentration of fascin. Hence, our observations suggest that GUV size and fascin concentration both contribute to fascin-mediated filopodia-like protrusions by increasing the chance of actin bundles interacting with one another in a confined environment. Our results are in agreement with the formation of packed fascin-actin bundles which remain straight at the tip of protrusion (Tsai and Koenderink, 2015). It has been suggested that membrane elasticity can contribute to actin bundle assembly and protrusion of branched actin networks at the membrane (Liu et al., 2008). This model and occasional aggregation of straight bundles in protrusion sites suggest a mechanism by which membrane protrusion is facilitated through a feedback mechanism where flexible lipid bilayer and fascin both contribute to protrusion at the bundle-membrane interface.

External control over bundle disassembly and the consequent reversal of GUV shape by light exposure reported here can lay the foundation for numerous studies on membrane morphology and the mechanics of network–membrane complex in membrane-encapsulated biopolymer networks. One future approach could be light-induced localized disassembly of reconstituted actin bundles and branched actin networks by targeted photo-illumination (Linsmeier et al., 2016). This will enable direct observation of actin-membrane interactions upon actin network disassembly in only selected regions of interest. By cellular reconstitution of membrane-associated actin networks in the presence of fascin and network disassembly by targeted illumination, one can study the dynamics of assembly/disassembly in filopodia-like structures, which can provide insights into the understanding of transition/remission from lamellipodia to filopodia in reconstituted networks (Haviv et al., 2006) and in living cells.

Our findings revealed that fascin-mediated filopodia-like protrusions in cell-sized GUVs were suppressed in the presence of α-actinin. We have shown recently that actin bundles are more likely to undergo transient interactions with the bilayer at high α-actinin concentrations (Bashirzadeh et al., 2020). Hence, the bending of encapsulated α-actinin-actin bundles and their interaction with the membrane is expected to be responsible for the inhibition of protrusion. Supported by coarse-grained simulations, bulk and motility reconstitution assays have demonstrated spontaneous sorting and domain formation of fascin and α-actinin in a crosslinker size-dependent manner (Winkelman et al., 2016; Freedman et al., 2019). As protein sorting is expected to contribute to the formation of filopodia by fascin-bundles, the mechanism of inhibition of membrane spikes in the presence of α-actinin remains an open question. Visualizing fluorescently labeled crosslinkers in actin bundles can potentially reveal the existence of such mechanism.

## Data Availability Statement

The raw data supporting the conclusions of this article will be made available by the authors, without undue reservation.

## Author Contributions

YB and AL designed the experiments. YB and NW conducted the experiments. YB analyzed the experimental data. YB, NW, and AL prepared purified proteins. YB developed the data processing methods. YB, NW, and AL wrote the manuscript. All authors discussed the results and commented on the manuscript.

## Conflict of Interest

The authors declare that the research was conducted in the absence of any commercial or financial relationships that could be construed as a potential conflict of interest.
