# Supplementary material for: Confinement Geometry Tunes Fascin-Actin Bundle Structures and Consequently the Shape of a Lipid Bilayer Vesicle
Source: Front Mol Biosci. 2020 Nov 9;7:610277. doi: 10.3389/fmolb.2020.610277 (PMC7680900; doi:10.3389/fmolb.2020.610277)
Supplement: Supplementary file 1 [file Data_Sheet_1.PDF]

Confinement geometry tunes fascin-actin bundle structures and consequently the shape of a lipid bilayer vesicle

Yashar Bashirzadeh[1], Nadab Wubshet[1], Allen P. Liu[1,2,3,4]

[1] Department of Mechanical Engineering, University of Michigan, Ann Arbor, Michigan, 48109, USA

[2] Department of Biomedical Engineering, University of Michigan, Ann Arbor, Michigan, 48109, USA

[3] Department of Biophysics, University of Michigan, Ann Arbor, Michigan, 48109, USA

[4] Cellular and Molecular Biology Program, University of Michigan, Ann Arbor, Michigan, 48109, USA

Corresponding author:

A.P.L: allenliu@umich.edu; 2350 Hayward Street, University of Michigan, Ann Arbor, Michigan, 48109, USA. Tel: +1 734-764-7719.

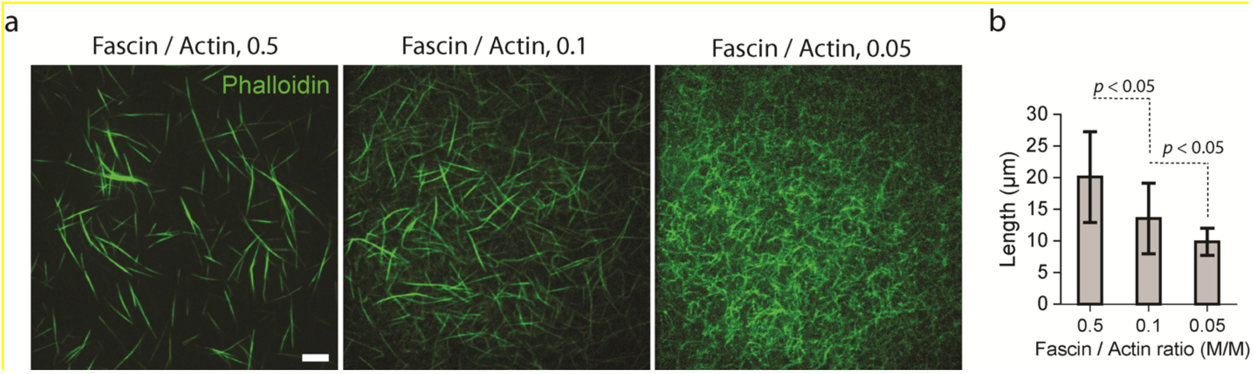

Supplemental Figure 1. a, Fluorescence confocal images of actin bundles stabilized with Acti-stain 488 phalloidin at molar ratios of fascin indicated. Actin, 5 µM. Scale bar, 10 µm. **b,** Length distribution of actin bundles in bulk at different molar ratios of fascin. Actin, 5 µM. Error bar indicates standard deviation. Number of bundles imaged were 55, 59, and 99 for fascin / actin molar ratios of 0.5, 0.1, and 0.05, respectively.

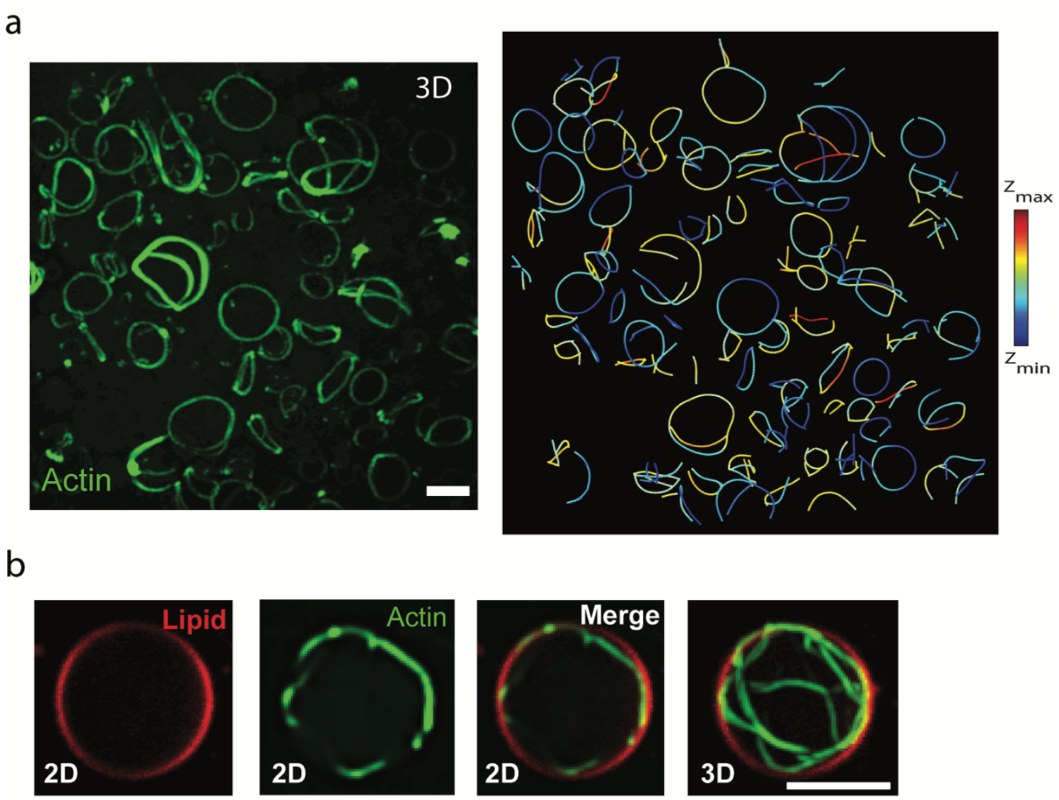

Supplemental Figure 2. a, 3D confocal (left) and skeletonized (right) image of a representative GUV population encapsulating α-actinin-actin bundles. **b,** 2D lipid, actin, merged, and 3D merged images of a representative small GUV encapsulating α-actinin-actin bundles bent around and co-localized with GUV membrane. α-actinin/actin, 0.1 (M/M). Actin, 5 µM. Scale bars, 10 µm.

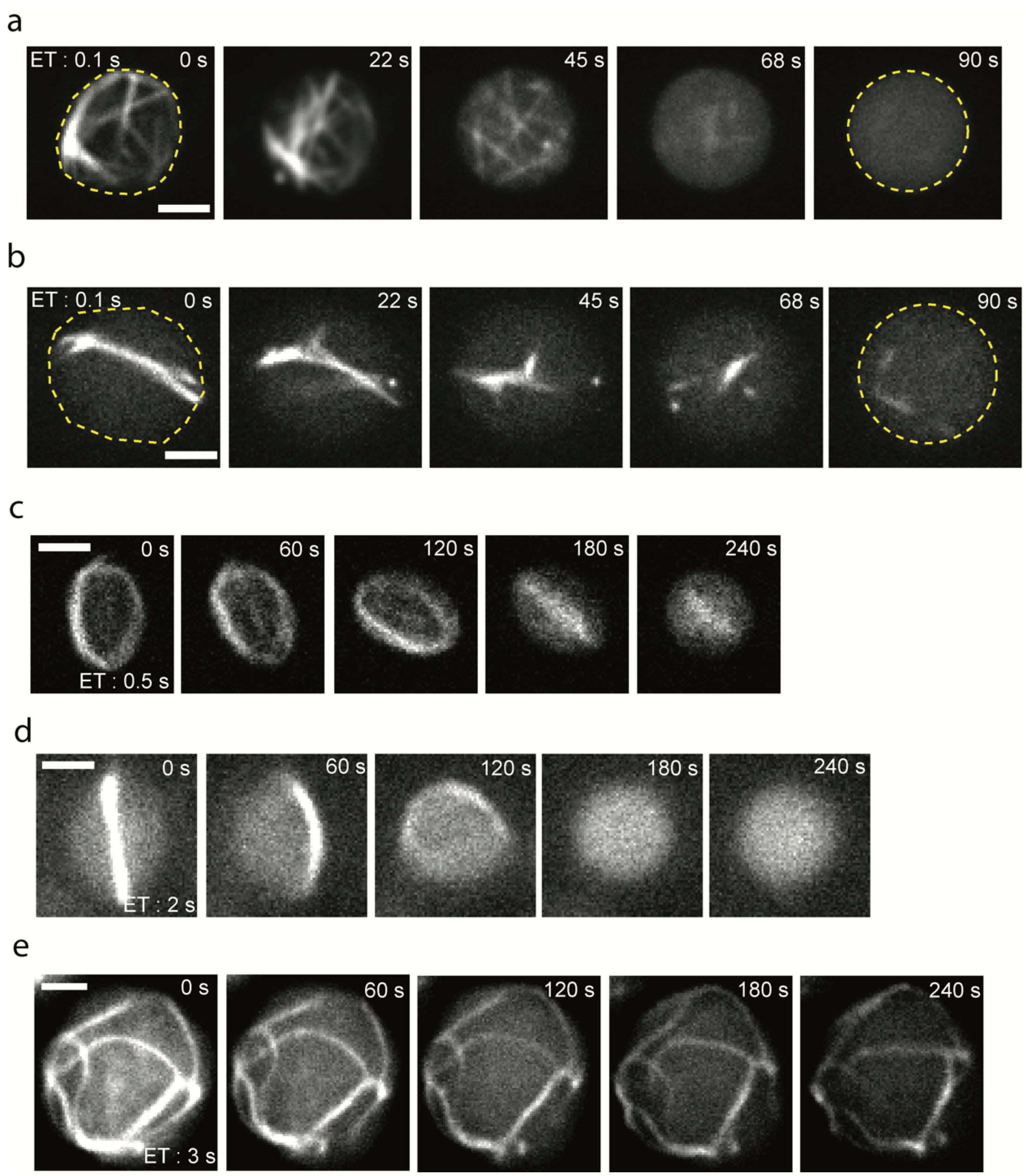

Supplemental Figure 3. a,b, Exposure to 488 nm light with 500 ms exposure time captured at 2 frames/s induces the disassembly of fascin-actin bundles and restoration of GUV shape in ≤ 90 s . Fascin/actin, 0.1 (M/M). Actin, 5 µM. Scale bars, 5 µm. **c,** Exposure to 488 nm light with 500 ms exposure time captured at 1 frame/2 s induces the disassembly of fascin-α-actinin-actin bundles and restoration of GUV shape in 4 min. fascin/actin, 0.1 (M/M). α-actinin/actin, 0.1 (M/M). Actin, 5 µM. Scale bar, 5 µm. **d,** Increasing α-actinin concentration required longer exposure time (2 s) for fascin-α-actinin-actin bundle disassembly. Images were captured at 1 frame/2 s. Fascin/actin 0.1 (M/M). α-actinin/actin, 0.2 (M/M). Actin, 5 µM. Scale bar, 5 µm. **e,** α-actinin-actin bundles did not disassemble even at high exposure times (3 s). Images were captured at 1 frame/3 s. α-actinin/actin, 0.2 (M/M). Actin, 5 µM. Scale bar, 5 µm.
