# Supplementary material for: Confinement Geometry Tunes Fascin-Actin Bundle Structures and Consequently the Shape of a Lipid Bilayer Vesicle
Source: Front Mol Biosci. 2020 Nov 9;7:610277. doi: 10.3389/fmolb.2020.610277 (PMC7680900; doi:10.3389/fmolb.2020.610277)
Supplement: Supplementary file 2 [file Data_Sheet_2.PDF]

Supplementary video captions

Supplemental Video 1. Application of 488 nm light at high exposure time (3 s) resulted in the disassembly of actin bundles and reversal of GUV shape without affecting the integrity of the lipid bilayer. Fascin/actin, 0.5 (M/M). Scale bar, 10 μm. Images were captured at 1 frame/3.6 s. Actin, green. Lipid, red.

Supplemental Video 2. Exposure to 488 nm light with 500 ms exposure time captured at 2 frames/s induces the disassembly of fascin-actin bundles and restoration of GUV shape in ≤ 90 s. The video is a time-lapse actin image sequence. Fascin/actin, 0.1 (M/M). Scale bar, 10 μm.

Supplemental Video 3. Exposure to 488 nm light with 500 ms exposure time captured at 2 frames/s induces the disassembly of an amalgamated single bundle and restoration of GUV shape. The video is a time-lapse actin image sequence. Fascin/actin, 0.1 (M/M). Scale bar, 10 μm.

Supplemental Video 4. Exposure to 488 nm light with 500 ms exposure time captured at 1 frame/2 s induces the disassembly of fascin-α-actinin-actin bundles and restoration of GUV shape in 4 min. The video is a time-lapse actin image sequence. Fascin/actin, 0.1 (M/M). α-actinin/actin, 0.1 (M/M). Scale bar, 10 μm.

Supplemental Video 5. Exposure to 488 nm light with 2 s exposure time captured at 1 frame/2 s induces the disassembly of fascin-α-actinin-actin bundles and restoration of GUV shape in 4 min. The video is a time-lapse actin image sequence. Fascin/actin, 0.1 (M/M). α-actinin/actin, 0.2 (M/M). Scale bar, 10 μm.

Supplemental Video 6. Exposure to 488 nm light with 3 s exposure time captured at 1 frame/3 s did not induce the disassembly of α-actinin-actin bundles. The video is a time-lapse actin image sequence. α-actinin/actin, 0.2 (M/M). Scale bar, 10 μm.
